# Exposures to Endocrine-Disrupting Chemicals and Age of Menarche in Adolescent Girls in NHANES (2003–2008)

**DOI:** 10.1289/ehp.1104748

**Published:** 2012-08-14

**Authors:** Danielle E. Buttke, Kanta Sircar, Colleen Martin

**Affiliations:** National Center for Environmental Health, Centers for Disease Control and Prevention, Atlanta, Georgia, USA

**Keywords:** 2,4-dichlorophenol, endocrine disruptors, menarche, NHANES, reproductive health

## Abstract

Background: The observed age of menarche has fallen, which may have important adverse social and health consequences. Increased exposure to endocrine-disrupting compounds (EDCs) has been associated with adverse reproductive outcomes.

Objective: Our objective was to assess the relationship between EDC exposure and the age of menarche in adolescent girls.

Methods: We used data from female participants 12–16 years of age who had completed the reproductive health questionnaire and laboratory examination for the Centers for Disease Control and Prevention’s National Health and Nutrition Examination Survey (NHANES) for years 2003–2008 (2005–2008 for analyses of phthalates and parabens). Exposures were assessed based on creatinine-corrected natural log urine concentrations of selected environmental chemicals and metabolites found in at least 75% of samples in our study sample. We used Cox proportional hazards analysis in SAS 9.2 survey procedures to estimate associations after accounting for censored data among participants who had not reached menarche. We evaluated body mass index (BMI; kilograms per meter squared), family income-to-poverty ratio, race/ethnicity, mother’s smoking status during pregnancy, and birth weight as potential confounders.

Results: The weighted mean age of menarche was 12.0 years of age. Among 440 girls with both reproductive health and laboratory data, after accounting for BMI and race/ethnicity, we found that 2,5-dichlorophenol (2,5-DCP) and summed environmental phenols (2,5-DCP and 2,4-DCP) were inversely associated with age of menarche [hazard ratios of 1.10; 95% confidence interval (CI): 1.01, 1.19 and 1.09; 95% CI: 1.01, 1.19, respectively]. Other exposures (total parabens, bisphenol A, triclosan, benzophenone-3, total phthalates, and 2,4-DCP) were not significantly associated with age of menarche.

Conclusions: Our findings suggest an association between 2,5-DCP, a potential EDC, and earlier age of menarche in the general U.S. population.

Over the past century, the average age of menarche has declined worldwide, from 16–17 years to < 13 years of age (Euling et al. 2008). This documented trend toward a younger age of menarche has been observed consistently across socioeconomic and race/ethnicity groups (Lee et al. 2001; Wronka 2010) and cannot be attributed to increased nutritional status alone (Himes et al. 2009; Wang 2002).

An earlier onset of menarche is associated with many adverse health and social outcomes (Zuckerman 2001). Earlier menarche has been associated with increased breast cancer risk (Iwasaki et al. 2007; Maskarinec et al. 2006; Stavraky and Emmons 1974), adult-onset asthma (Al-Sahab et al. 2010; Macsali et al. 2011), shorter adult stature (Carel 2006; Partsch and Sippell 2001), hyperinsulinemia and metabolic syndrome (Frontini et al. 2003), type 2 diabetes (He et al. 2010), and reproductive tract cancers (Dossus et al. 2010; Elwood et al. 1977; Fujita et al. 2008). Furthermore, decreased age of menarche has been associated with behavioral and psychosocial disorders, as well as with increased risk of sexual abuse, depression, and teen pregnancy (Deardorff et al. 2005; Jacobson-Dickman and Lee 2009; Joinson et al. 2011; Phinney et al. 1990).

Scientists have proposed many theories to explain the progressive decline in age of onset of menarche (Zuckerman 2001). No consistent relationship between age of menarche and smoking status, socioeconomic status, family structure, or formula feeding has been seen (Rokade and Mane 2009). Another hypothesis regarding the cause of earlier puberty and menarche is increased environmental exposure to endocrine-disrupting compounds (EDCs) in household and personal care products (Buck Louis et al. 2008; Chen et al. 2011; Ozen et al. 2012; Schell and Gallo 2010). EDCs are synthetic or natural compounds that can mimic, alter, or attenuate the action of natural hormones found in the body.

Several compounds to which the general U.S. population is regularly exposed have been implicated as affecting endocrine function or development. For example, certain benzophenones, dichlorophenols, parabens, and the compound triclosan have been shown to disrupt estrogen receptor signaling in animal and *in vitro* models either by binding directly to the receptor itself or through modulation of downstream signaling processes (Akahori et al. 2008; Craig et al. 2011; Kawaguchi et al 2009; Shaw and deCatanzaro 2009; Stoker et al. 2010; Vo et al. 2010; Yamasaki et al. 2005). Conversely, compounds such as phthalates and bisphenol A have been shown in human and animal studies to disrupt androgen-dependent processes (Howdeshell et al. 2008; Miao et al 2011; Svechnikov et al. 2010), with bisphenol A implicated in both anti-androgenic and estrogenic responses (Chao et al. 2012). Each of these chemicals is manufactured at high volumes for use in household products such as plastic food containers, personal care products, and household cleaners and deodorizers, allowing potentially high exposures to occur in the general public through daily activities and behaviors.

Our objective was to use the 2003–2008 National Health and Nutrition Examination Survey (NHANES) data to assess the potential association between environmental exposures to synthetic EDCs, as assessed by urinary biomarkers, and age of menarche after adjusting for various demographic and health-related factors. We evaluated chemicals previously identified as potential endocrine disruptors that are found in most of the U.S. population and were measured in NHANES participants during at least 3 consecutive years.

## Methods

*Study population*. NHANES is a cross-sectional, multistage, stratified, cluster sampling survey conducted by the Centers for Disease Control and Prevention’s (CDC) National Center for Health Statistics. NHANES uses a complex sampling design that includes questionnaires about demographics and health-related behaviors, as well as laboratory and clinical measurements (CDC 2011). We limited our analysis to participants between 12 and 16 years of age to capture exposures close to the age of menarche among those who completed reproductive heath questionnaires (administered beginning at age 12). Reproductive health questionnaires were administered through a proxy of parent or guardian to females 12 years of age, the lower age limit in our analysis, to 16 years. All procedures were approved by the NCHS Institutional Review Board, and all participants provided written informed consent. During 2003–2004, NHANES sampled urinary phenols and phthalates in two separate one-third subsets, and a single one-third subset was analyzed for phenols, phthalates, and parabens from 2005 through 2008. In each case, subsets were a representative sample of NHANES participants ≥ 6 years of age from each 2-year study cycle. Therefore, data presented for phenols and phthalates are from years 2003–2008, whereas data from parabens are from years 2005–2008 only. Laboratory samples were collected on the same day the questionnaire was administered.

*Measurement of urinary phenols, parabens, and phthalates.* Phenols and parabens. Environmental phenols considered for inclusion were bisphenol A (BPA), 4-*tert*-octyl phenol, 2,4,4´-trichloro-2´-hydroxyphenyl ether (triclosan), 2-hydroxy-4-metoxybenzophenone (benzophenone-3). Parabens considered for inclusion were propyl-, butyl-, ethyl-, and methyl-paraben, 2,4-dichlorophenol (2,4-DCP), *o*-phenyl phenol, 2,5-dichlorophenol (2,5-DCP), 2,4,5-trichlorophenol, and 2,4,6-trichlorophenol (CDC 2011). These substances were quantified in urine by use of solid phase extraction (SPE) coupled on-line to high performance liquid chromatography–tandem mass spectrometry (HPLC-MS/MS) (Ye et al. 2005, 2006). In addition we evaluated the phenol metabolites 2,4-DCP, *o*-phenyl phenol, 2,5-DCP, 2,4,5-trichlorophenol, and 2,4,6-trichlorophenol, which were measured in urine by use of SPE-HPLC followed by atmospheric pressure chemical ionization–high-performance liquid chromatography–isotope dilution tandem mass spectrometry (APCI-MS/MS) (Ye et al. 2005).

Phthalate metabolites. We used HPLC-MS/MS with electrospray ionization to quantify the following phthalate metabolites in urine: monomethyl phthalate, monoethyl phthalate, monobutyl phthalate, mono-isobutyl phthalate, mono(3-carboxypropyl) phthalate, monocyclohexyl phthalate, mono(2-ethylhexyl) phthalate, monooctyl phthalate, monobenzyl phthalate, monoisononyl phthalate, mono(2-ethyl-5-oxohexyl) phthalate, mono(2-ethyl-5-hydroxyhexyl) phthalate, mono(2-ethyl-5-carboxypentyl) phthalate, monocarboxyoctyl phthalate, and monocarboxynonyl phthalate (CDC 2011).Before analysis by HPLC-MS/MS, the urine samples were processed through use of enzymatic deconjugation of the glucuronidated phthalate monoesters, followed by on-line SPE (Kato et al. 2005; Silva et al. 2007).

*Data analysis.* We included female 2003–2008 NHANES study participants 12–16 years of age who had completed the reproductive health questionnaire and physical examination, and for whom data regarding age of menarche, defined by NHANES as age of first menstruation, were available. Of the 1,598 individuals 12–16 years of age who had completed the reproductive health questionnaire, 1,420 participants had complete data on body mass index (BMI) and age of menarche (age or not yet reached). Of these, 461 were included in NHANES subsamples with urinary phenol and phthalate measurements. We used the one-third subsample weighting variables for each 2-year subset of data. Nonmissing values for urine concentrations below the limit of detection (LOD) were replaced with the value of the LOD divided by the square root of 2. In our analysis, all urinary compounds and metabolites were creatinine-corrected by dividing urine concentrations by creatinine concentrations to give micrograms per gram of creatinine as the final units. Urine samples with creatinine levels > 300 mg/dL or < 30 mg/dL were excluded because they were too dilute or too concentrated for accurate analysis (*n* = 11) (Sata et al. 1995).

We used SAS 9.2 for data analysis, and calculated means and percentiles of the EDCs and demographic factors by use of the PROC SURVEYMEANS (weighted geometric means) procedure to account for the complex sampling design of NHANES. We calculated the weighted mean self-reported age of menarche using PROC LIFETEST (Kaplan–Meier censored survival estimates) to account for censoring at the age of participation among 43 individuals who had not reached menarche at the time of participation (all programs from SAS Institute Inc., Cary, NC). We used the Taylor series (linearization) method to estimate standard errors and confidence intervals. We calculated BMI percentile for age in months using the standardized CDC growth charts (Grummer-Strawn et al. 2010). We set significance at α = 0.05 for two-sided *p*-values.

To estimate associations between EDCs and age of menarche, we used the PROC SURVEYPHREG (Cox-proportional hazards model with censored data) procedure and the efron method of ties handling, after confirming the proportional hazards assumption. We modeled the natural log of creatinine-corrected urine analyte concentrations to normalize distributions in our data and, in accordance with NHANES analytic guidelines, we excluded 10 observations with outlier values > 3 SDs for deviance residuals of the log-transformed weighted values, as determined by a probability plot of residuals (CDC 2011). These adjustments left a sample size of 440 for our phenol analysis, 437 for phthalate analysis, and 287 individuals for parabens analysis.

We assessed the urinary concentration of 18 EDCs found above the LOD in at least 75% of study participants as exposures in our model, out of a total of 27 phthalate, phenol, and pesticide compounds or metabolites evaluated in urine in NHANES. These compounds were then analyzed either as single compounds (benzophenone-3, triclosan, BPA, 2,4-DCP, and 2,5-DCP) or as the sum of urinary analyte concentrations within a class of compounds (parabens, phthalates, and environmental phenols), again including only those compounds found above the LOD in at least 75% of study participants. We converted compounds to molar weights for summing to adjust for the different molecular weights of compounds and used micrograms per gram of creatinine as the unit in our models. We identified potential confounders from the literature (mother’s age at birth of girl, mother’s smoking status during pregnancy, birth weight < 5.5 pounds (yes or no), birth weight > 9.0 pounds (yes or no), family income (1–12 categories based on NHANES categories of $0–4,999 to ≥ $75,000), family income-to-poverty ratio (0–5), self-defined race/ethnicity, BMI (continuous) as calculated by measurements taken during the NHANES examination, and BMI percentile for sex and age in months (0–100) as calculated using CDC standardized growth charts) (Grummer-Strawn et al. 2010) and included them in our final models if they either predicted the outcome with *p* < 0.05 or there was a > 10% change in the hazard ratio for the exposure–menarche association when they were removed from the model. Insufficient observations existed for birth weight to include in model building. Backward and forward selection resulted in the same final model.

We evaluated BMI and race/ethnicity as effect modifiers by evaluating stratum-specific hazard ratios because differences in age of menarche among ethnicities are well established (Himes et al. 2009). Interactions between race/ethnicity and urinary phenol concentrations were evaluated using interaction terms in the model, with *p* < 0.10 for the interaction term used to evaluate significance of the interaction.

## Results

Most participants included in our analysis (63.1%) were of non-Hispanic white ethnicity (Table 1). Of the 342 with complete information on mother’s smoking status, 13.9% had mothers who smoked during pregnancy. The mean family income-to-poverty ratio was 2.6 [95% confidence interval (CI): 2.4, 2.7], the mean mother’s age at the participant’s birth was 26.8 years (95% CI: 26.3, 27.3), the mean BMI was 22.8 (95% CI: 22.2, 23.4), and the average age of menarche was 12.0 years (95% CI: 11.8, 12.1). Age of menarche ranged from 8 to 16 years, with 43 individuals not having attained menarche by the time of survey.

**Table 1:** Descriptive statistics for age at menarche and selected covariates among girls 12–16 years of age in NHANES 2003–2008.

Variable	No. of participants	Weighted percent^a^ or mean value (95% CI)
Race/ethnicity (%)
Non-Hispanic white	124	63.1 (55.4, 70.7)
Non-Hispanic black	135	14.6 (10.8, 18.4)
Mexican American	135	11.9 (8.1, 15.7)
Other Hispanic	25	5.2 (2.3, 8.2)
Other, multiracial	21	5.2 (2.3, 8.1)
Smoking status of mother during pregnancy (%)
Yes	33	13.9 (8.9, 18.5)
No	309	86.1 (81.5, 91.1)
Family income-to-poverty ratio	440	2.6 (2.4, 2.7)
Mother’s age at participant’s birth (years)	347	26.8 (26.3, 27.3)
Self-reported age at menarche (years)	440	12.0 (11.8, 12.1)
BMI (kg/m2)b	440	22.8 (22.2, 23.4)
aEstimated percent distribution (95% CI) after applying NHANES sampling weights. bBMI as measured by the mobile examination clinic personnel.				

Mean BMI was higher among non-Hispanic black girls than among non-Hispanic white girls and girls of other, multiracial ethnicities (Table 2). The mean age of menarche was significantly lower among non-Hispanic black adolescent girls than among non-Hispanic white adolescent girls.

**Table 2:** Weighted mean BMI and self-reported age of menarche by race/ethnicity among girls 12–16 years of age in NHANES 2003–2008 (*n* = 440).

Race/ethnicity	*n*	Weighted mean BMI (95% CI)	Weighted mean BMI percentile (95% CI)	Weighted mean age of menarche^a^ (95% CI)
Non-Hispanic white	124	22.1 (21.3, 22.9)	59.6 (53.6, 65.6)	12.1 (11.9, 12.3)
Non-Hispanic black	135	25.5 (24.7, 26.4)	76.6 (72.2, 81.0)	11.5 (11.3, 11.7)
Mexican American	135	23.2 (21.9, 24.4)	67.6 (62.2, 73.0)	11.8 (11.6, 12.0)
Other Hispanic	25	25.2 (22.4, 28.0)	77.0 (62.2, 91.9)	11.4 (10.7, 12.1)
Other, multi-racial	21	19.9 (18.1, 21.7)	48.5 (34.3, 64.7)	11.9 (11.7, 12.2)
Total	440	22.8 (22.2, 23.4)	63.3 (59.2, 67.5)	12.0 (11.8, 12.1)
aAge of menarche is self-reported as age in years of first menstrual period.								

Three phenols, BPA, benzophenone-3, and triclosan, were each above the LOD in at least 75% of study participants and were evaluated as single compounds in our analysis. The parabens propyl paraben and methyl paraben were above the LOD in at least 75% of study participants and were summed as total parabens for analysis. The phthalate metabolites monoethyl phthalate, monobutyl phthalate, mono-isobutyl phthalate, mono(3-carboxypropyl) phthalate, mono(2-ethylhexyl) phthalate, monobenzyl phthalate, mono(2-ethyl-5-oxohexyl) phthalate, mono(2-ethyl-5-hydroxyhexyl) phthalate, mono(2-ethyl-5-carboxypentyl) phthalate, monocarboxyoctyl phthalate, and monocarboxynonyl phthalate were above the LOD in at least 75% of study participants and were summed as total phthalates for analysis. The phenol metabolites 2,4-DCP and 2,5-DCP were above the LOD in 99.8% and 91.8% of study participants, respectively, and were evaluated both as separate compounds and summed as total phenol metabolites. Geometric means and 95% CIs of urinary creatinine-adjusted EDC levels are reported in Table 3.

**Table 3:** Estimated creatinine-corrected weighted geometric means (µg/g creatinine) of selected EDCs and EDC metabolites among girls 12–16 years of age in NHANES 2003–2008 by race/ethnicity.

Analyte	Geometric mean (µg/g) (95% CI)										
	Non-Hispanic white (*n* = 124)	Non-Hispanic black (*n* = 135)	Mexican American (*n* = 135)	Other Hispanic (*n* = 25)	Other, multiracial (*n* = 21)	Total (*n* = 440)
BPA (n = 440)	2.47 (2.14, 2.86)	2.22 (1.91, 2.57)	1.85 (1.5, 2.2)	1.48 (1.20, 1.84)	1.80 (1.23, 2.64)	2.25 (2.02, 2.52)
Benzophenone-3 (n = 440)	36.5 (24.3, 54.8)	12.3 (9.62, 15.7)	18.7 (14.3, 24.4)	18.7 (10.0, 34.8)	27.4 (11.1, 67.7)	27.4 (21.6, 36.8)
Triclosan (n = 440)	13.6-(8.44, 21.9)	6.92 (4.89, 9.80)	10.3 (6.44, 16.6)	9.48 (3.92, 22.9)	47.4 (10.1, 223)	12.4 (8.98, 17.2)
Total parabensa (n = 287)	593 (410, 858)	1,360 (1,030, 1,790)	859 (637, 1,157)	277 (161, 476)	535 (179, 1,600)	668 (527, 847)
Total phthalate metabolitesb (n = 437)	1.61 (1.35, 1.92)	1.76 (1.48, 2.10)	1.89 (1.57, 2.29)	1.99 (1.48, 2.67)	1.57 (1.20, 2.06)	1.68 (1.49, 1.90)
2,4-DCP (n = 440)	0.637 (0.520, 0.787)	1.15 (0.850, 1.55)	1.21 (0.90, 1.62)	0.678 (0.380, 1.22)	0.927 (0.550, 1.57)	0.766 (0.660, 0.890)
2,5-DCP (n = 440)	5.89 (4.55, 7.62)	25.0 (15.7, 39.7)	19.6 (12.3, 31.1)	13.3 (6.54, 27.1)	14.7 (7.20, 30.2)	9.18 (7.49, 11.3)
Total phenol metabolitesc (n = 440)	7.07 (5.53, 9.03)	26.8 (17.0, 42.0)	21.7 (13.9, 33.7)	14.8 (7.46, 29.4)	16.1 (8.13, 31.9)	10.6 (8.78, 12.9)
aTotal parabens include propyl paraben and methyl paraben. bTotal phthalate metabolites include phthalate metabolites monoethyl phthalate, monobutyl phthalate, mono-isobutyl phthalate, mono(3-carboxypropyl) phthalate, mono(2-ethylhexyl) phthalate, monobenzyl phthalate, mono(2-ethyl-5-oxohexyl) phthalate, mono(2-ethyl-5-hydroxyhexyl) phthalate, mono(2-ethyl-5-carboxypentyl) phthalate, monocarboxyoctyl phthalate, and monocarboxynonyl phthalate. cTotal phenol metabolites include 2,4-DCP and 2,5-DCP.												

Consistent with observations from the complete NHANES data set, the mean environmental phenol concentration differed by race/ethnicity, with non-Hispanic white girls (*n* = 124; mean = 7.07 µg/g; 95% CI: 5.53, 9.03) having statistically significantly lower environmental phenol concentrations than non-Hispanic black (*n* = 136, mean = 26.8 µg/L; 95% CI: 17.0, 42.0) on the basis of nonoverlapping CIs (Table 3). Concentrations of other compounds did not differ significantly by race/ethnicity (data not shown).

Family income-to-poverty ratio, mother’s age at the participant’s birth, and the mother’s smoking status during pregnancy were not retained in our model because they were not significant predictors of menarche.

Total parabens, BPA, triclosan, benzophenone-3, and total phthalates were not significantly associated with age of menarche, either before or after adjustment for BMI and race/ethnicity (Table 4). However, a 1-unit increase in log-transformed creatinine-corrected urine phenol concentration was associated with a 0.7-month decrease in average age of menarche (adjusted hazard ratio = 1.10; 95% CI: 1.02, 1.20; *p* = 0.01). 2,5-DCP was the only individual compound that was significantly associated with age of menarche (adjusted hazard ratio = 1.10; 95% CI: 1.01, 1.19; *p* = 0.025). The association (hazard ratios) between urinary 2,5-DCP or total environmental phenol concentration and age of menarche did not differ by race/ethnicity (data not shown).

**Table 4:** Weighted survival analysis model estimates and parameters of time of menarche and log creatinine-corrected urinary concentrations (µg/g creatinine) of potential EDCs among girls 12–16 years of age in NHANES 2003–2008 participating in the reproductive health questionnaire and environmental phenol laboratory analysis.

Parameter	Pr > |t|^a^	Unadjusted HR (95% CI)	HR adjusted for race/ethnicity (95% CI)	HR adjusted for BMI and race/ethnicity (95% CI)	HR adjusted for BMI percentile and race/ethnicity (95% CI)
Total parabens^b^ (*n* = 287)	0.47	1.06 (0.94, 1.18)	1.04 (0.92, 1.17)	0.96 (0.84, 1.09)	1.05 (0.93, 1.19)
BPA (n = 441)	0.50	0.93 (0.78, 1.11)	0.97 (0.82, 1.15)	0.94 (0.79, 1.13)	0.94 (0.80, 1.10)
Triclosan (n = 440)	0.50	0.96 (0.88, 1.04)	0.96 (0.90, 1.04)	0.97 (0.90, 1.05)	1.0 (0.91, 1.09)
Benzophenone-3 (n = 440)	0.62	0.95 (0.88, 1.02)	0.98 (0.90, 1.06)	0.98 (0.89, 1.07)	0.99 (0.91, 1.08)
Total phthalatesc (n = 437)	0.39	0.92 (0.79, 1.08)	0.91 (0.77, 1.07)	0.95 (0.85, 1.07)	0.98 (0.86, 1.12)
Total environmental phenold (n = 440)	0.017	1.10 (1.02, 1.18)	1.07 (0.99, 1.16)	1.10 (1.02, 1.20)	1.10 (1.01, 1.19)
2,5-DCP (n = 440)	0.025	1.15 (1.07, 1.23)	1.13 (1.04, 1.23)	1.10 (1.01, 1.19)	1.09 (1.01, 1.19)
2,4-DCP (n = 440)	0.066	1.06 (0.98, 1.15)	1.04 (0.96, 1.12)	1.08 (0.99, 1.18)	1.02 (0.94, 1.13)
HR, hazard ratio. aModel was adjusted by BMI and race/ethinicity. bTotal parabens includes propyl paraben and methyl paraben. cTotal phthalate metabolites include phthalate metabolites monoethyl phthalate, monobutyl phthalate, mono-isobutyl phthalate, mono (3-carboxypropyl) phthalate, mono(2-ethylhexyl) phthalate, monobenzyl phthalate, mono(2-ethyl-5-oxohexyl) phthalate, mono(2-ethyl-5-hydroxyhexyl) phthalate, mono(2-ethyl-5-carboxypentyl) phthalate, monocarboxyoctyl phthalate, and monocarboxynonyl phthalate. dTotal phenol metabolites include 2,4-DCP and 2,5-DCP.										

When we considered the total environmental phenol concentration for adolescent girls above the 75th percentile, the average age of menarche was 11.8 years (95% CI: 11.6, 12.0). In comparison, the average age of menarche for adolescent girls below the 25th percentile of total environmental phenol urine concentration was 12.4 years (95% CI: 12.2, 12.6) (Table 5).

**Table 5:** Weighted mean self-reported age of menarche by percentile of creatinine-corrected urinary environmental phenol concentration in girls 12–16 years of age in NHANES 2003–2008 (*n* = 440).

Percentile of phenol concentration of study participantsa	Weighted geometric mean creatinine-corrected total phenol concentration (µg/g creatinine) (95%CI)	Weighted geometric mean creatinine-corrected 2,5‑DCP concentration (µg/g creatinine) (95%CI)	Weighted mean age (years) of menarche (95% CI)
< 25th		2.36 (2.14, 2.60)		3.21 (2.84, 3.63)		12.4 (12.2, 12.6)
25th– < 50th		7.39 (6.74, 8.10)		8.06 (6.95, 9.34)		12.2 (12.0, 12.4)
50th < 75th		26.7 (24.3, 29.8)		27.5 (23.2, 32.4)		11.7 (11.5, 11.9)
≥ 75th		207 (156, 275)		196 (142, 270)		11.8 (11.6, 12.0)
aPercentiles determined based on total phenol concentration.						

## Discussion

Levels of urinary EDCs in our study population were comparable to levels reported in the 6–11 and 12–19 years age groups in the overall 2005–2008 NHANES population (CDC 2011).

A significant, inverse relationship was seen between age of menarche and urinary environmental phenols and 2,5-DCP. Girls with urinary environmental phenol concentrations above the 75th percentile had a significantly lower age of menarche than girls below the 75th percentile. No other significant associations were seen between urinary EDC biomarkers and age of menarche. The difference in age of menarche between those in the highest percentile of exposure and those in the lowest percentile of exposure was small and may not be clinically significant, but if the association was causal it suggests a physiologic effect.

We found an association between 2,5-DCP, as assessed by urinary biomarkers, and age of menarche in a representative sample of the U.S. population. Another cross-sectional study reported an association between serum PBDEs (polybrominated diphenyl ethers) and age of menarche in adolescent girls (Chen et al. 2011). Three cohort studies have examined relationships between EDC biomarkers and early menarche. A study of 151 women living in the Great Lakes region of the United States reported an association between *in utero* exposure to the DDT (dichlorodiphenyltrichloroethane) metabolite DDE (dichlorodiphenyldichloroethylene) and early menarche (Vasiliu et al. 2004). Among a cohort of Michigan women exposed to polybrominated biphenyl (PBB) flame retardants from contaminated dairy and beef there was an association between *in utero* PBB exposure and early menarche among their female children (Blanck et al. 2000). In a retrospective study, serum DDT levels were associated with early puberty in a retrospective cohort study of children in Belgium, but the association was limited to 145 children who were born outside of Belgium, and confounding by factors related to place of birth or related factors could not be ruled out (Krstevska-Konstantinova et al. 2001). Blood lead levels have been associated with delayed puberty in boys and girls (Hauser et al. 2008; Selevan et al. 2003), and exposures to dioxins have been associated with delayed puberty in boys (Korrick et al. 2011).

2,5-DCP is the major metabolite of dichlorobenzene (DCB), a common fumigant also used in mothballs, insect repellants, deodorizers, and toilet bowl disinfectants. 2,5-DCP can be used to estimate exposure to the putative endocrine disruptor DCB in the previous 6–12 months, which is particularly important when interpreting an end point such as age of menarche that is dependent on earlier changes in the neuroendocrine axis of the brain (Teitelbaum et al. 2008). DCB is found in drinking water at concentrations up to 1 mg/L and in food at levels up to 10 mg/kg (Barber et al. 2009). The U.S. population was exposed to a nearly constant level of 25 µg/m^3^ DCB in indoor air, according to a longitudinal study of homes in 1987 (Wallace 1991a, 1991b; Wallace and Pellizzari 1995), and, although air concentrations may have declined since these studies, DCB was still readily detected in amniotic fluid (Bradman et al. 2003) and human breast milk at concentrations of 5–30 mg/kg nearly 20 years after the indoor air study (Ye et al. 2006).

Like benzophenones, phenols, and parabens, DCB has been reported to have both *in vitro* EDC activity and EDC effects in laboratory animals [Darbre and Harvey 2008; Fang et al. 2000; Hershberger et al. 2005; Makita 2008; Marsman 1995; Molina-Molina et al. 2008; National Toxicology Program (NTP) 2008; Takahashi et al. 2011; Versonnen et al. 2003]. The levels of DCB at which EDC activity was evident were consistent with levels observed for human daily exposures as well as at higher levels of exposure. DCB has been associated with low birth weight and low maternal weight gain in rats (Marsman 1995) and sperm abnormalities in male mice and rats (Takahashi et al. 2011). In a study of 404 pregnant women, urinary levels of 2,5-DCP during the third trimester of pregnancy were associated with low birth weight and length in male humans (Wolff et al. 2008). DCB is hepatotoxic and hepatocarcinogenic to rats and mice (NTP 1987, 2002), and it reduces plasma levels of thyroxine in rats (den Besten et al. 1991). DCB is currently undergoing tier 1 testing for endocrine-disrupting potential by the U.S. Environmental Protection Agency (2010).

The present study has several limitations. First, questionnaire data are self-reported and may be subject to misclassification. Second, we cannot rule out potential confounding by unmeasured factors associated with DCB exposure and the timing of puberty and menarche. In addition, exposures were measured at a single point in time after the onset of menarche in all but 43 participants; ideally, we would measure urinary EDCs several times during the 6–36 months before menarche, and adjust for BMI before menarche only. Age of menarche was recorded as age in years at first menstrual period. This limitation is further complicated by the half-life of these compounds. Although the U.S. population is continuously exposed to compounds such as BPA, dichlorophenols, and certain phthalates, resulting in relatively constant levels of these compounds in serum and urine (CDC 2011), the relatively short half-lives of these compounds limits our ability to predict exposures before menarche using a single urine sample. Differences in urine 2,5-DCP concentrations also may reflect differences in metabolism, rather than direct differences in individual exposures. Finally, we cannot rule out chance findings due to multiple comparisons, and it is possible that the association between 2,5-DCP and decreased age of menarche could reflect changes in personal habits, pharmacokinetics, or metabolism after menarche.

## Conclusions

We estimated an inverse association between urinary 2,5-DCP concentration and age of menarche in girls 12–16 years of age who participated in the NHANES study during 2003–2008. To our knowledge, ours is the first population-based study to report an association between exposure to the putative environmental EDC dichlorobenzene and age of menarche, an outcome that may reflect endocrine-disrupting effects. Although this finding must be interpreted with caution given study limitations, it highlights the need for more research into the potential role of environmental exposures to potential EDCs and adverse endocrine and reproductive health outcomes in humans.
